# The Effects of Environmental Sustainability Labels on Selection, Purchase, and Consumption of Food and Drink Products: A Systematic Review

**DOI:** 10.1177/0013916521995473

**Published:** 2021-02-20

**Authors:** Christina Potter, Anastasios Bastounis, Jamie Hartmann-Boyce, Cristina Stewart, Kerstin Frie, Kate Tudor, Filippo Bianchi, Emma Cartwright, Brian Cook, Mike Rayner, Susan A. Jebb

**Affiliations:** 1 University of Oxford, UK; 2 University of Nottingham, UK

**Keywords:** systematic review, ecolabels, food, demand

## Abstract

This review assessed the effects of environmental labels on consumers’ demand for more sustainable food products. Six electronic databases were searched for experimental studies of ecolabels and food choices. We followed standard Cochrane methods and results were synthesized using vote counting. Fifty-six studies (*N* = 42,768 participants, 76 interventions) were included. Outcomes comprised selection (*n* = 14), purchase (*n* = 40) and consumption (*n* = 2). The ecolabel was presented as text (*n* = 36), logo (*n* = 13) or combination (*n* = 27). Message types included: organic (*n* = 25), environmentally sustainable (*n* = 27), greenhouse gas emissions (*n* = 17), and assorted “other” message types (*n* = 7). Ecolabels were tested in actual (*n* = 15) and hypothetical (*n* = 41) environments. Thirty-nine studies received an unclear or high RoB rating. Sixty comparisons favored the intervention and 16 favored control. Ecolabeling with a variety of messages and formats was associated with the selection and purchase of more sustainable food products.

## Introduction

There is an urgent need to move toward healthier and more sustainable diets in order to feed a growing population within planetary boundaries (Willett et al., 2019). One potentially promising avenue to change individuals’ dietary choices is through the use of environmental sustainability labels (hereafter: ecolabels). Ecolabels are defined as information or claims provided with a product that tell consumers about the quality, features or production methods that reduce environmental impact, aiming to facilitate informed decision-making (Thøgersen et al., 2010).

Ecolabels are yet to be commonplace, but there are a number of reasons to think they may help shift consumer behavior. Nutrition and health labeling on foods is now widely implemented, with research showing such labels lead to small changes in purchasing and consumption behaviors, for example by reducing the energy content in food and drinks purchased and increasing purchasing of items with health-related claims (Crockett et al., 2018). Preliminary evidence suggests ecolabeling may be a means of meeting societal demands for greater transparency in reporting food production methods (D’Amico et al., 2016).

A recent review of the factors affecting consumer “green” purchasing behavior highlights that ecolabels may have potential to change behavior and increase demand for more environmentally sustainable products (Joshi & Rahman, 2015) but the evidence on ecolabels has yet to be systematically synthesized. There is a large diversity in the type of information conveyed and the contexts within which ecolabels are presented, both of which may impact effectiveness (Ibanez, 2016). For example, ecolabels promoted by different stakeholders, for example, environmental organizations, governments, multi-national, and/or domestic firms may influence consumer perceptions of value and trustworthiness of the label (Ibanez, 2016). In addition, different consumer groups may respond to ecolabels in different ways (Teisl et al., 2008).

We aimed to systematically review the effect of ecolabels on the selection, purchase and/or consumption of more environmentally sustainable food and drink products, both in real and hypothetical (e.g., online experimental supermarket platform) environments. We also aimed to assess whether any effect of ecolabels is moderated by the presentation format, the type of information being presented, and/or the presence of a certification label, and whether effects vary by socio-demographic characteristics (e.g., gender, level of education).

## Method

The protocol for this systematic review was published in advance and is registered on PROSPERO (Ref: PROSPERO ID CRD42018087635). We followed PRISMA guidelines to report the findings (Moher et al., 2009). The methods for searching, screening, data extraction and risk of bias (RoB) assessment followed those described in the updated Cochrane Handbook for synthesizing results using a non-meta-analytic approach (McKenzie & Brennan, 2019).

### Searching and Inclusion Criteria

We searched six electronic databases (1973–present) using terms related to food labeling, environment, and choice behavior (date of most recent search 20 April 2019) (see protocol PROSPERO ID CRD42018087635 for full strategy; Supplemental Table 1 in the online Appendix for MEDLINE search strategy). We included studies that were designed to measure effects of ecolabels on the selection, purchase and/or consumption of any foods or drinks in both actual and hypothetical (e.g., online experimental supermarket platform) environments. Studies were eligible if they followed an experimental intervention design, including multi-arm designs. Studies were ineligible if they only used non-experimental or qualitative methods. Studies examining the effects of social responsibility ecolabels (e.g., Fairtrade Mark), animal welfare ecolabels (e.g., “Dolphin Safe”) or genetic modification labels (e.g., GMO-Free) were excluded. No geographical region was excluded. However, we only included studies where the full-text was written in English. For a full list of inclusion and exclusion criteria, please refer to the review protocol (PROSPERO ID CRD42018087635).

### Screening, Data Extraction, and Risk of Bias Assessment

Studies were screened by two independent reviewers for inclusion at title/abstract and full-text stage, with disagreements resolved by discussion or referral to a third reviewer. Using a predefined and piloted data extraction form, including an adapted version of the Cochrane risk-of-bias tool (Higgins et al., 2011; Kaur et al., 2017), study data were extracted in duplicate and inconsistencies were resolved through discussion or referral to a third reviewer. Data were extracted on inclusion/exclusion criteria; population; setting (real or hypothetical); intervention and comparator characteristics; outcomes (selection, purchase, and/or consumption); and whether these varied by gender or socioeconomic status.

Study quality was assessed across the following potential sources of bias: random sequence generation; allocation sequence concealment; blinding of participants and personnel; blinding of outcome assessment; incomplete outcome data (e.g., attrition); selective outcome reporting; or other biases. Studies that received at least one high-risk rating in any of the individual categories of the RoB were given an overall high-risk rating and studies with at least one unclear-risk rating and no high-risk ratings in any of the individual categories of the RoB were given an overall unclear RoB rating. Only studies that received low-risk ratings across all individual categories on the RoB tool were given a low overall RoB rating.

### Analysis

Due to substantial clinical heterogeneity, statistical synthesis was not possible. As recommended by Cochrane, we used a vote-counting method based on direction of effect and tabulated results for our primary and secondary outcomes using effect direction plots (McKenzie & Brennan, 2019). We classified data by outcome:

- Selection: whether participants select a food or drink product; no money is exchanged.
- Purchase: whether participants purchase a food or drink product; money is exchanged. Purchasing may be measured at the individual or store level and money may be participants’ own or provided to the participant by the researcher.
- Consumption: whether participants consume a food or drink product.

Results are presented separately for real and hypothetical settings. We classified “real” settings as those in which actual food or drink products were selected, purchased (with real money) or consumed. We classified “hypothetical” settings as those in which participants could hypothetically select or purchase products, without actually being given the product or spending real money. Additionally, interventions were classified based on the following categories:

- Information versus Claims: Information was classified as detail about the product itself (or its production) which is typically shown as a value (e.g., “creates 50 kg CO_2_,” “uses 50 gallons of water”) whereas a claim was typically based on a broader judgement, sometimes including unquantified metrics (e.g., “environmentally sustainable,” “uses less water than the alternative,” or “Organic”). The distinctions made between information and claims were based on the taxonomy of health-related food labeling for the International Network for Food and Obesity/NCD research, Monitoring and Action Support (INFORMAS) (Rayner et al., 2013).
- Presentation format: logo (visual), text, or both.
- Content type: Greenhouse Gas (GHG) emissions, Organic, Environmentally Sustainable (or similar), or Other (including ecolabels regarding land use, water use, or pesticide use). Studies which tested sustainability labels or claims, such as “Sustainably harvested,” “Ecologically friendly,” “Sustainably managed,” were included and categorized as “Environmentally Sustainable” labels.

## Results

### Search and Screening

After removing duplicates, 2,624 references were retrieved from database searches. Two independent reviewers assessed the full text of 361 studies, 305 of which were excluded because the study did not measure the primary outcomes of interest or followed a non-experimental design. After screening, we included 55 references, representing 56 studies and 76 relevant interventions (see Supplemental Figure S1 in the online Appendix for a PRISMA flow diagram).

#### Participants and settings of included studies

This review includes 42,768 participants. Twenty-nine studies were conducted in Europe, 16 were conducted in North America, seven in Asia, two in Australia, one in South America, and one was conducted in different countries across continents. Further details of included studies can be found in Table 1.

**Table 1.** Characteristics of Included Studies.

Study ID	Country	% Females	Message type	Overall RoB
Aerni et al. (2011)	Switzerland	NR	Organic	Unclear
Aguilar et al. (2010)	USA	60.0%	Organic, other (pesticide-free)	High
Aizaki et al. (2013)	Japan	72.4%	Environmentally sustainable	Low
Akaichi et al. (2016)	Scotland, France, & the Netherlands	NR	Organic, GHG emissions	Unclear
Ankamah-Yeboah et al. (2018)	Germany	60.3%	Organic, ASC certified	High
Aoki and Akai (2013)	Japan	NR	GHG emissions	High
Aoki et al. (2017)	Japan & Thailand	62.4% (Thailand) & 49.9% (Japan)	Organic	Unclear
Apaolaza et al. (2017)	Spain	34.0%	Organic	Unclear
Aprile et al. (2012)	Italy	63.0%	Organic	High
Bauer et al. (2013)	Germany	54.3%	Organic	Low
Blend and Van Ravenswaay (1999)	USA	77.0%	Environmentally sustainable	High
Borin et al. (2011)	USA	NR	Other (pesticide use)	High
Brach et al. (2018)	Germany	58.1%	Organic, environmentally sustainable	High
Brayden et al. (2018)	USA	52.3%	Organic, environmentally sustainable	High
Brunner et al. (2018)	Sweden	30.0%	GHG emissions	High
Campbell-Arvai et al. (2014)	USA	52.7%	Environmentally sustainable	Unclear
Caputo et al. (2018)	Belgium	63.0%	Organic, environmentally sustainable	Low
Carlsson et al. (2010)	Sweden	44.0%	Organic, environmentally sustainable	Low
Carlucci et al. (2017)	Italy	55.4%	Organic	Low
Cho (2014)	USA	38.4%	Environmentally sustainable	High
Cho and Baskin (2018)	USA	39.6%	Environmentally sustainable	Low
Cholette et al. (2013)	USA	51.0%	GHG emissions	Unclear
Cosmina et al. (2016)	Italy	62.0%	Organic	Unclear
Daunfeldt and Rudholm (2014)	Sweden	NR	Organic	Unclear
Delmas and Lessem (2017)	USA	47.6%	Organic	Low
De Pelsmacker et al. (2005)	Belgium	59.3%	Environmentally sustainable	High
Durham et al. (2012)	USA	70.6% (Minnesota), 60.3% (Portland), 67.4% (Rhode Island)	Environmentally sustainable	Unclear
Elofsson et al. (2016)	Sweden	NR	GHG emissions	Low
Fernandez-Polanco et al. (2013)	Spain	78.0%	Environmentally sustainable	High
Gosselt et al. (2019)	The Netherlands	55.0%	Environmentally sustainable	Low
Grebitus et al. (2013)	Canada	52.0%	GHG emissions, Other (water use)	High
Grunert et al. (2014)	UK, France, Germany, Spain, Sweden & Poland	UK: 50.8%, France: 50.6%, Germany: 55%, Spain: 47.5%, Poland: 50.8%, Sweden: 50.7%	GHG emissions	Unclear
Gumirakiza et al. (2017)	USA	61.9%	Organic	High
Hallstein and Villas-Boas (2013)	USA	NR	Environmentally sustainable	Low
Harwood and Drake (2018)	USA	NR	Organic, environmentally sustainable	High
Hoek et al. (2017)	Australia	65.0%	Environmentally sustainable	Low
Hoogland et al. (2007)	The Netherlands	67.0%	Organic	Low
Jaffry et al. (2004)	UK	NR	Environmentally sustainable	Low
Kim et al. (2013)	USA	NR	Organic	High
Lee et al. (2018)	Taiwan	64.8%	Organic	High
Lee et al. (2018)	Taiwan	74.0%	Organic	High
Mondelaers et al. (2009)	Belgium	NR	Organic	High
Osman and Thornton (2019)	UK	70% (exp 1), 33.4% (exp 2)	Environmentally sustainable, GHG emissions	High
Panzone et al. (2011)	UK	62.2%	GHG emissions	High
Pelletier et al. (2016)	Australia	NR	GHG emissions	High
Peschel et al. (2016)	Canada & Germany	52.0% (C), 55.0% (G)	GHG emissions, other (water use)	High
Risius et al. (2017)	Germany	65.0%	Organic, environmentally sustainable	Low
Rokka c (2008)	Finland	62.0%	Environmentally sustainable	High
Seo et al. (2019)	Japan	100.0%	Organic, other (pesticide use)	Unclear
Shuai et al. (2014)	China	58.1%	GHG emissions	High
Silva et al. (2017)	Brazil	70.0%	Environmentally sustainable, organic	High
Vlaeminck et al. (2014)	Belgium	54.0%	GHG emissions, Other (water use, land use)	Low
Wessells et al. (1999)	USA	NR	Environmentally sustainable	High
Wuepper et al. (2019)	Germany	51.0%	Other (water use)	Low
Zanoli et al. (2015)	Italy	70.3%	Organic	Low

*Note*. NR = not reported; GHG = greenhouse gas; RoB = risk of bias.

#### Characteristics of the included interventions

The majority of the studies included in this review (41 out of 56) were conducted in hypothetical settings. All studies followed an experimental design with 30 conducting some type of discrete choice experiment (DCE) (see Tables 4–6). In 35 studies the intervention constituted a claim, 14 studies provided information only, and seven studies reported a mixed intervention type. In 10 studies, the intervention was presented in a logo format, in 29 studies as text and 17 studies used a mixed format. In 16 studies an organic ecolabel was tested, eight studies tested a GHG emissions ecolabel, 14 studies tested an environmentally sustainable ecolabel, and two studies tested an “other” type of ecolabel (e.g., pesticide use, water use). In 15 studies ecolabels with mixed claims and/or information were tested, including different combinations of organic, GHG emissions, environmentally sustainable and other ecolabels. In 38 studies a food product was used, in 14 studies a drink product was used, and in four studies both food and drink products were used.

Certification schemes were present in the experimental conditions of 29 studies (Tables 4–6). Certified labels have undergone some method, either through internal or third-party assessments, to verify the label validity. In 20 studies an organic label was accompanied by a known certification scheme, classified as: (i) international (e.g., USDA, EU, Biogarantie, CCPB, or DEMETER); (ii) national; (iii) local; (iv) private brands/certification schemes; and (v) organic certifications depicting products that adhered to certified organic quality control systems. In nine studies environmental sustainability labels were underpinned by a published certification scheme such as the Agricultural Stewardship Council (i.e., ASC) (Ankamah-Yeboah et al., 2018; Risius et al., 2017), Good Agricultural Practice (GAP) program (Aizaki et al., 2013), World Wildlife Foundation (WWF), and local Marine Stewardship Council (MSC) (Wessells et al., 1999), certifications based on different farming practices (Durham et al., 2012), CO_2_ emissions (Elofsson et al., 2016), or different sustainable harvesting practices (Brayden et al., 2018).

#### Risk of bias

Overall, 28 studies received a high RoB rating, 11 studies received an unclear RoB rating, and 17 studies received a low RoB rating (Table 1). Studies judged to be at high RoB were not associated with larger effects (17 of 28 high RoB studies favored intervention; 8 of 11 unclear RoB studies favored intervention; 16 of 17 low RoB studies favored intervention). Most of the high and unclear RoB ratings in the individual categories of the RoB pertained to methods related to allocation concealment and blinding of the conditions in which participants were being tested. Most of the studies included in this review followed a DCE design in which a formal randomization procedure is not possible. Random sequence generation and allocation concealment were therefore rated as non-applicable for these studies. Studies that used some form of masking received a low-risk rating in blinding. Table 2 lists judgments by RoB domain for individual studies.

**Table 2.** Risk of Bias Judgments by Domain for Individual Studies.

Study ID	Random sequence generation	Allocation sequence concealment	Blinding of participants and personnel	Blinding of outcome assessment	Incomplete outcome data (e.g., attrition)	Selective outcome reporting	Other biases	Overall RoB
Aerni et al. (2011)	N/A	N/A	Low	Low	N/A	Low	Low	Unclear
Aguilar et al. (2010)	Unclear	Unclear	High	High	N/A	Low	Low	High
Aizaki et al. (2013)	N/A	N/A	Low	Low	Low	Low	Low	Low
Akaichi et al. (2016)	N/A	N/A	Unclear	Low	N/A	N/A	Low	Unclear
Ankamah-Yeboah et al. (2018)	Low	N/A	N/A	Low	N/A	High	Low	High
Aoki and Akai (2013)	N/A	N/A	Low	Low	Low	High	Low	High
Aoki et al. (2017)	N/A	Unclear	Low	Low	N/A	Low	Unclear	Unclear
Apaolaza et al. (2017)	N/A	N/A	Low	Low	N/A	N/A	Unclear	Unclear
Aprile et al. (2012)	Unclear	Unclear	High	High	N/A	Low	Low	High
Bauer et al. (2013)	Low	Low	Low	Low	N/A	Low	Low	Low
Blend and Van Ravenswaay (1999)	Low	Unclear	High	High	N/A	Low	Low	High
Borin et al. (2011)	High	High	N/A	N/A	N/A	Low	Low	High
Brach et al. (2018)	Low	N/A	Low	Low	N/A	High	Low	High
Brayden et al. (2018)	N/A	N/A	Low	Low	N/A	High	Low	High
Brunner et al. (2018)	High	High	Low	Low	Low	Low	Low	High
Campbell-Arvai et al. (2014)	N/A	N/A	Low	Low	N/A	Low	Unclear	Unclear
Caputo et al. (2018)	Low	Low	Low	Low	N/A	Low	Low	Low
Carlsson et al. (2010)	N/A	N/A	Low	Low	N/A	Low	Low	Low
Carlucci et al. (2017)	Low	Low	Low	Low	N/A	Low	Low	Low
Cho (2014)	Low	Low	Unclear	Unclear	High	Low	Low	High
Cho and Baskin (2018)	N/A	Low	Low	Low	N/A	Low	Low	Low
Cholette et al. (2013)	N/A	N/A	Low	Low	N/A	Low	Unclear	Unclear
Cosmina et al. (2016)	N/A	N/A	Low	Low	N/A	Unclear	Low	Unclear
Daunfeldt and Rudholm (2014)	N/A	N/A	N/A	N/A	N/A	Unclear	Low	Unclear
Delmas and Lessem (2017)	N/A	N/A	Low	Low	N/A	Low	Low	Low
De Pelsmacker et al. (2005)	N/A	N/A	Unclear	Unclear	N/A	High	Low	High
Durham et al. (2012)	N/A	N/A	Unclear	Unclear	N/A	Low	Low	Unclear
Elofsson et al. (2016)	N/A	N/A	Low	Low	N/A	Low	Low	Low
Fernandez-Polanco et al. (2013)	N/A	N/A	High	High	N/A	Unclear	Low	High
Gosselt et al. (2019)	Low	N/A	Low	Low	Low	Low	Low	Low
Grebitus et al. (2013)	Low	Low	Unclear	Unclear	N/A	High	Low	High
Grunert et al. (2014)	N/A	N/A	Unclear	Unclear	Low	Low	Low	Unclear
Gumirakiza et al. (2017)	Low	Low	Unclear	Unclear	N/A	High	High	High
Hallstein and Villas-Boas (2013)	N/A	N/A	Low	Low	N/A	Low	Low	Low
Harwood and Drake (2018)	N/A	N/A	Low	Low	N/A	High	Low	High
Hoek et al. (2017)	N/A	Low	Low	Low	Low	Low	Low	Low
Hoogland et al. (2007)	N/A	N/A	Low	Low	N/A	Low	Low	Low
Jaffry et al. (2004)	N/A	N/A	Low	Low	N/A	Low	Low	Low
Kim et al. (2013)	Low	N/A	Low	Low	N/A	Unclear	Unclear	High
Lee et al. (2018)	Low	Unclear	Low	Low	Unclear	Low	High	High
Lee et al. (2018)	Low	Low	Low	Low	Unclear	Low	High	High
Mondelaers et al. (2009)	N/A	N/A	High	High	N/A	Low	Unclear	High
Osman and Thornton (2019)	Low	Low	High	High	N/A	Low	Low	High
Panzone et al. (2011)	Low	Low	Low	Low	N/A	Low	High	High
Pelletier et al. (2016)	N/A	N/A	High	High	N/A	Low	High	High
Peschel et al. (2016)	Unclear	Unclear	High	High	N/A	Low	Low	High
Risius et al. (2017)	Low	Low	Low	Low	N/A	Low	Low	Low
Rokka and Uusitalo (2008)	N/A	N/A	Low	Low	N/A	High	Unclear	High
Seo et al. (2019)	Unclear	N/A	Unclear	Unclear	N/A	Low	Low	Unclear
Shuai et al. (2014)	N/A	N/A	High	High	N/A	High	High	High
Silva et al. (2017)	Low	Unclear	High	High	Low	High	High	High
Vlaeminck et al. (2014)	Low	Low	Low	Low	N/A	Low	Low	Low
Wessells et al. (1999)	Unclear	Low	High	High	High	High	High	High
Wuepper et al. (2019)	Low	Low	N/A	Low	N/A	Low	Low	Low
Zanoli et al. (2015)	N/A	N/A	Low	Low	N/A	Low	Low	Low

*Note*. RoB = risk of bias.

#### Outcomes

Four studies assessed actual selection, 10 studies assessed hypothetical selection, 10 studies assessed actual purchase, 30 studies assessed hypothetical purchase, one study assessed actual consumption and one assessed hypothetical consumption (Tables 4–6). Table 3 provides the percentages of comparisons which favored the intervention condition grouped by label type (GHG emissions, Organic, Environmentally Sustainable, or Other) and format (information vs claim; logo, text, or both). If a study is listed as having “mixed results” this means that a study tested the effects of an ecolabel across multiple food and/or drink products and found both positive and negative effects across trial arms.

**Table 3.** Comparisons Favoring the Intervention (%) Grouped by Label Type and Format.

	Information			Claim		
	Logo	Text	Both	Logo	Text	Both
GHG emissions	% of comparisons favoring the intervention: 50%, number of interventions: 2	% of comparisons favoring the intervention: 50%, number of interventions: 6	% of comparisons favoring the intervention: 100%, number of interventions: 6	% of comparisons favoring the intervention: 50%, number of interventions: 2	N/A	% of comparisons favoring the intervention: 100%, number of interventions: 1
Organic	N/A—organic labels were classified as claims not information			% of comparisons favoring the intervention: 100%, number of interventions: 4	% of comparisons favoring the intervention: 91%, number of interventions: 11	% of comparisons favoring the intervention: 80%, number of interventions: 10
Environmentally sustainable	N/A	% of comparisons favoring the intervention: 100%, number of interventions: 5	% of comparisons favoring the intervention: 0%, number of interventions: 3	% of comparisons favoring the intervention: 100%, number of interventions: 5	% of comparisons favoring the intervention: 67%, number of interventions: 9	% of comparisons favoring the intervention: 60%, number of interventions: 5
Other	N/A	% of comparisons favoring the intervention: 100%, number and type of interventions: 4, pesticide free (1), water use (2), land use (1)	N/A	N/A	% of comparisons favoring the intervention: 100%, number and type of interventions: 1, pesticide free (1)	% of comparisons favoring the intervention: 100%, number and type of interventions: 2, water use (2)
Totals across information and claim interventions	Logo format: 13 total, of which 11 favored intervention (85% effective)					
	Text format: 36 total, of which 29 favored intervention (81% effective)					
	Combined text and logo format: 27 total, of which 20 favored intervention (74% effective)					

**Table 4.** Effect Direction Plot of the Included Studies Grouped by Outcome and Label Type.

Study ID	Study design	Sample size	Product type	Presentation format (logo or text)	Intervention format (info or claim	Certification scheme	Supporting hypothesis	SS
Outcome: actual selection								
Label type: environmentally sustainable								
Campbell-Arvai et al. (2014)	CE	320	Meat-free items	L+T	I	N	Mixed◀▶	N
Jaffry et al. (2004)	CE	600	Seafood	T	I	Y	Y -▲	Y
Label tyle: GHG emissions								
Cholette et al. (2013)	Survey	428	Apples	T	I	N	NR	NR
Label type: mixed								
Aguilar et al. (2010)	CBCA	524	Chestnuts	T	C + I, I: pesticide free; C: organic	Y	Y -▲	Y
Outcome: hypothetical selection								
Label type: organic								
Aprile et al. (2012)	CE	200	Olive oil	L	C	Y	Y -▲	Y
Cosmina et al. (2016)	CE	420	Coffee	T	C	Y	Y -▲	Y
Label type: environmentally sustainable								
Carlsson et al. (2010)	CE	768	Coffee	T	C	N	Y -▲	Y
De Pelsmacker et al. (2005)	Web-based experimental study	750	Coffee	T	C	N	N -▼	Y
Osman and Thornton (2019)	Experimental study	417	Main meals	T	I	N	Y -▲	N
Wessells et al. (1999)	Contingent choice survey	1,640	Salmon, cod, and cocktail shrimp	L+T	C+I	Y	N -▼	NR
Label type: mixed								
Ankamah-Yeboah et al. (2018)	Online survey	610	Trout	L+T	C: organic; ASC certified	Y	Y -▲	Y
Brayden et al. (2018)	Online experimental study	2,155	Seafood	T	C: organic; certified sustainably harvested	Y	Y -▲	Y
Grunert et al. (2014)	Online choice experiment	4,408	Chocolate, coffee, ice cream, breakfast cereal, ready meals and soft drinks	L	C: rainforest alliance certified; carbon footprint	Y	Y -▲	Y
Peschel et al. (2016)	CE	3,130	Ground beef & potatoes	T	I GHG emissions; water use	N	Y -▲	Y

*Note*. L = logo; T = text; C = claim; I = information; NR = not reported; SS = statistically significant; GHG = greenhouse gas; ▲ = statistically significant positive effect; ▲ = not statistically significant positive effect, ▼ = statistically significant negative effect; ▼ = not statistically significant negative effect; ◀▶ = both statistically non-significant positive and negative effects observed (mixed result).

**Table 5.** Effect Direction Plot of the Included Studies Grouped by Outcome and Label Type.

Study ID	Study design	Sample size	Product type	Presentation format (logo or text)	Intervention format (info or claim	Certification scheme	Supporting hypothesis	SS
Outcome: actual purchase								
Label type: organic								
Aerni et al. (2011)	Experimental study	3275	Corn bread	T	C	N	Y -▲	Y
Daunfeldt and Rudholm (2014)	Natural experiment	NR	Olive oil, flour, coffee	L	C	Y	Y -▲	Y
Zanoli et al. (2015)	CE	427	Apples	L	C	Y	Y -▲	Y
Label type: GHG emissions								
Aoki and Akai (2013)	CE	212	Oranges	T	I	N	Y -▲	Y
Brunner et al. (2018)	Field experiment	2524	Meat, fish, salads	L	I	N	Mixed◀▶	Y
Elofsson et al. (2016)	Field experiment	NR	Milk	L+T	C+I	Y	Y -▲	Y
Pelletier et al. (2016)	Experimental study	NR	Various products	L	I	N	Y -▲	Y
Label type: environmentally sustainable								
Hallstein and Villas-Boas (2013)	Quasi-experimental study	NR	Seafood	L	C	N	Y -▲	Y
Label type: mixed								
Vlaeminck et al. (2014)	Field experiment	150	Food market products	L+T	I: GHG emissions; water use; land use	N	Y -▲	Y
Wuepper et al. (2019)	CE	NR	Coffee	L+T	C: organic; water use	N	Y -▲	Y
Outcome: hypothetical purchase								
Label type: organic								
Aoki et al. (2017)	CE	3,395	Rice	T	C	Y	Y -▲	Y
Apaolaza et al. (2017)	Experimental study	90	Wine	L+T	C	N	Y -▲	Y
Bauer et al. (2013)	Online experiment	630	Cereals	L+T	C	Y	Y -▲	Y
Carlucci et al. (2017)	CE	800	Oysters	T	C	Y	Y -▲	Y
Delmas and Lessem (2017)	DCE	883	Wine	T	C	Y	Y -▲	Y
Gumirakiza et al. (2017)	CE	819	Peaches, eggplants, and yellow squash	T	C	Y	Y -▲	Y
Harwood and Drake (2018)	ACBC	1,163	Milk	T	C	Y	Y -▲	Y
Hoogland et al. (2007)	Field experimental study	371	Chicken, milk, salmon	L	C	Y	Y -▲	Y
Kim et al. (2013)	lab acceptance test	208	Milk	T	C	N	Y -▲	NR
Mondelaers et al. (2009)	choice preference experiment	529	Carrots	T	C	N	N -▼	N
Silva et al. (2017)	Pre-post experimental study	126	Dark chocolate	L+T	C	Y	Y -▲	NR
Label type: environmentally sustainable								
Aizaki et al. (2013)	CE	624	Milk	L	C	Y	Y -▲	Y
Blend and Van Ravenswaay (1999)	Experimental study	893	Apples	T	C	Y	Mixed◀▶	Y
Brach et al. (2018)	Online experiment	101	Various food products	T	C	Y	N -▼	Y
Cho (2014)	Experimental study	203	Apple pie and frozen pizza	T	I	N	Y -▲	Y
Cho and Baskin (2018)	Experimental study	53	Cereals & canned sausage	T	I	N	Y -▲	Y
Durham et al. (2012)	Experimental study	1500	Coffee	T	C+I	Y	Y -▲	Y
Fernandez-Polanco et al. (2013)	DCE	169	Seabream	T	C	N	Y -▲	Y
Gosselt et al. (2019)	Experimental study	180	Coffee	L+T	C	N	Y -▲	Y
Hoek et al. (2017)	CE	944	Rice, meat, tomato	L+T	C+I	N	N -▼	N
Risius et al. (2017)	CE	447	Trout	L+T	C	Y	Y -▲	Y
Rokka and Uusitalo (2008)	CBCA	330	Functional drinks	T	C	Y	Y -▲	Y
Label type: GHG emissions								
Panzone et al. (2011)	Experimental study	1,377	Cola, milk, meat, butter	T	I	N	N -▼	N
Shuai et al. (2014)	CE	873	Agri-food products	L	C	N	NR	NR
Label type: other claim (pesticide use)								
Borin et al. (2011)	Online experiment	329	Apples	T	I	N	Y -▲	N
Label type: mixed								
Akaichi et al. (2016)	CE	399	Bananas	L+T	C+I, C: organic; I: carbon footprint	Y	Y -▲	Y
Caputo et al. (2018)	CE	257	Chicken	L+T	C+I, C: organic; I: carbon footprint	Y	Y -▲	Y
Grebitus et al. (2013)	CE	1,551	Ground beef	T	I: GHG emissions; water use	N	Y -▲	Y
Seo et al. (2019)	CBCA	173	Spinach	T	C: organic; Pesticide-free	N	Y -▲	Y
Wuepper et al. (2019)	CE	NR	Coffee	L+T	C: organic; water use	N	Y -▲	Y

*Note*. L = logo; T = text; C = claim; I = information; NR = not reported; SS = statistically significant; GHG = greenhouse gas; ▲ = statistically significant positive effect; ▲ = not statistically significant positive effect; ▼ = not statistically significant negative effect; ◀▶ = both statistically significant positive and negative effects observed (mixed result).

**Table 6.** Effect Direction Plot of the Included Studies Grouped by Outcome and Label Type.

Study ID	Study design	Sample size	Product type	Presentation format (logo or text)	Intervention format (info or claim	Certification scheme	Supporting hypothesis	SS
Outcome: actual consumption								
Label type: organic								
Lee et al. (2018)	Lab experimental study	271	Raisins, chocolate balls	L+T	C	Y	N -▼	N
Outcome: hypothetical consumption								
Label type: organic								
Lee et al. (2018)	Online experimental study	122	Cookies	L+T	C	Y	Mixed◀▶	Y

*Note*. L = logo; T = text; C = claim; SS = statistically significant; GHG = greenhouse gas; ▼ = not statistically significant negative effect; ◀▶ = both statistically significant positive and negative effects observed (mixed result).

Across the 76 interventions, 17 assessed a GHG emissions ecolabel, 25 assessed an organic ecolabel, 27 assessed an environmentally sustainable ecolabel, and seven assessed other types of ecolabels (detailed in Table 3). All ecolabel formats were found to be effective in the majority of studies; 85% of comparisons in logo-only format favored intervention, 81% of text-only format favored intervention, and 74% of combined text and logo format ecolabels favored intervention.

For environmental sustainability messages, comparisons testing information presented in text-only form (five studies) and claims presented in logo-only form (five studies) consistently favored the intervention. The combination of logo and text formats appeared less effective when presenting environmentally sustainable information (not effective in any of the three interventions) or environmentally sustainable claims (effective in three of the five interventions).

Conversely, presenting GHG emissions information or claims using a combined logo and text format was the most effective approach for information (all six comparisons favored intervention) and claims (the one eligible comparison favored the intervention). Among logo-only and text-only formats for presenting GHG information or claims, there was a positive result favoring the intervention in only 50% of the 10 comparisons.

For organic claims, all formats were largely effective (logo-only: all four comparisons favored intervention; text-only: 10 of 11 comparisons favored intervention; combined text and logo: 8 of 10 comparisons favored intervention). “Other” ecolabel claims and/or information were evaluated in seven interventions and in all cases favored the intervention.

### Effects of Interventions Compared with Control Conditions

#### Selection

Of the four studies that tested actual selection, two found effects in favor of the intervention. One displayed information explaining the product contained minimal chemicals as a certified organic claim in text form (Aguilar et al., 2010), the other displayed environmentally sustainable information in text form (Jaffry et al., 2004). One study, which displayed environmentally sustainable information in logo and text form, found mixed effects (Campbell-Arvai et al., 2014). In one study we could not determine overall effect direction (Cholette et al., 2013). This study provided GHG emissions information in text form and attempted to identify characteristics of consumer segments and how their selection of ecolabeled items related to price (price considerations will be explored further in our companion review, PROSPERO ID: CRD42018094330).

Of the 10 studies that tested a hypothetical selection of products, eight studies found an effect favoring the intervention (see Table 4). Of the studies that found effects in favor of the intervention, two tested organic claims, one in logo (Aprile et al., 2012) and one in text form (Cosmina et al., 2016). Another two tested environmentally sustainable ecolabels, one as a claim in text form (Carlsson et al., 2010) and one as information in text form (Osman & Thornton, 2019). Four additional studies found effects in favor of the intervention when comparing one or more interventions (see Comparative Effectiveness).

Two studies of environmentally sustainable ecolabels found effects favoring the control condition. One provided an environmentally sustainable claim in text form (De Pelsmacker et al., 2005), and the other tested three types of ecolabels for seafood and found effects in favor of control (Wessells et al., 1999).

Across all studies examining selection behavior (actual and hypothetical), eight studies applied a certification scheme and positive effects were observed in six of these. The remaining study did not report data pertaining to the certification scheme (Wessells et al., 1999) (Table 4).

#### Purchase

Of the 10 studies that tested the effects on actual purchases, nine studies significantly favored the intervention, and one study (GHG emissions information in logo form) showed mixed effects across products (Brunner et al., 2018). The studies that favored the intervention condition included three organic claim interventions (two in logo and one in text form) (Aerni et al., 2011; Daunfeldt & Rudholm, 2014; Zanoli et al., 2015), three GHG emissions information interventions (one in text form, one in logo form, and one in combined logo and text form) (Aoki & Akai, 2013; Elofsson et al., 2016; Pelletier et al., 2016), one intervention assessing an environmentally sustainable claim in logo form (Hallstein & Villas-Boas, 2013), and one intervention assessing a water use claim in logo form (Wuepper et al., 2019).

Studies testing hypothetical purchase (*N* = 30) showed a similar pattern, with 24 studies favoring the intervention condition, four studies favoring the control condition, one study which provided an environmentally friendly claim (in text format) finding mixed effects across conditions and products (Blend & Van Ravenswaay, 1999), and one study in which we were unable to determine an overall direction of effect (Shuai et al., 2014) (Table 5).

Of the studies that tested an organic claim (*N* = 11) which favored the intervention (*N* = 10), six were in text form only, one in logo form only and three were in both text and logo form (see Table 5). Additionally, of those that provided environmentally sustainable labels which favored the intervention, five were in text form only, one was in logo form only, and two were in both text and logo form (see Table 5). Finally, one study that tested a pesticide use message in text form found effects in favor of the intervention (Borin et al., 2011). Another five studies found effects in favor of the intervention when comparing one or more interventions. These are described in more detail in the Comparative Effectiveness section.

Four studies found effects in favor of the control condition. Of these, one study provided an organic claim in text form (Mondelaers et al., 2009), one an environmentally sustainable claim in text form (Brach et al., 2018), one an environmentally sustainable claim and information in both logo and text form (Hoek et al., 2017), and another provided GHG emissions information in text form (Panzone et al., 2011). One study provided mixed results using an environmentally sustainable claim in text form (Blend & Van Ravenswaay, 1999). Additionally, in one study, we were unable to determine an overall direction of effect because purchasing behavior was examined in the context of consumer demographics; this study found that the hypothetical purchasing of low-carbon impact products (green carbon logos) was higher among men with higher incomes and higher levels of education (Shuai et al., 2014). Finally, across all studies examining purchasing behavior (actual or hypothetical), 19 applied a certification scheme. Positive effects were observed in the majority (16 out of 19) of these studies (Table 5).

#### Consumption

One paper reported two studies testing the effect of an organic ecolabel on food consumption (one study measured hypothetical consumption and the other measured actual consumption) (Lee et al., 2018). The hypothetical consumption experiment followed a 2 (“vice” vs. “virtue” food) × 2 (organic vs. unlabeled) experimental study design. Participants were asked to indicate how much they would eat if given the opportunity. This study found mixed effects of the organic label on hypothetical consumption. The laboratory-based experiment measured whether consumption of “vice” or “virtue” foods varied by the presence of an ecolabel (an organic label based on a certification scheme presented in combined text and logo form). There was no significant main effect of the organic label on actual consumption (Table 6).

### Comparative Effectiveness

Eleven studies directly compared two or more eligible interventions, two in actual and nine in hypothetical environments, and all found positive outcomes in favor of the ecolabel intervention. One study, which assessed actual selection, compared pesticide-free and organic claims and found no difference in effectiveness between the two (Aguilar et al., 2010). Another study, which assessed actual purchase behavior, compared two formats of information labels referring to GHG emissions, water use and land use. One format that combined a standardized color scale at the attribute level and a total environmental friendliness score at the product level was deemed the “most accessible” while the label providing raw information at the attribute level was the “least accessible.” This study found an effect in favor of the “most accessible” intervention (Vlaeminck et al., 2014).

Four studies compared multiple interventions assessing hypothetical selection behavior. The first study compared effects of certified organic claims (displayed in combined text and logo form) with the general Aquaculture Stewardship Council (ASC) claim (displayed in combined text and logo form), and found that respondents with lower income and higher age were more likely to prefer products with an organic label, but not with an ASC label (Ankamah-Yeboah et al., 2018). The second study, which compared the effects of organic and “certified sustainably harvested” claims in text form, found that people were equally likely to select products with either of these ecolabels compared to unlabeled products (Brayden et al., 2018). The third study compared effects of the Rainforest Alliance logo and the Carbon Footprint logo, finding that people were more likely to hypothetically select products with the Rainforest Alliance logo (Grunert et al., 2014). The fourth study compared effects of GHG emissions labels (in three levels; low, medium and high) with water usage labels (also in three levels; low, medium, and high), both in text form, on product selection. A multinomial logit model found that all choice attributes of the model (price, carbon, and water footprint) were significant (Peschel et al., 2016).

Five studies compared multiple interventions and assessed hypothetical purchase behavior. The first study examined possible trade-offs consumers make between organic claims (organic vs. not organic) and carbon footprint labels in logo form (with four levels of GHG emissions). Organic products and products with lower carbon footprint were preferred to non-organic products and products with a higher carbon footprint (Akaichi et al., 2016). A second study tested the effectiveness of a certified organic logo (organic vs. not organic) with a carbon footprint label (20% carbon footprint reduction vs. 30% carbon footprint reduction), finding no difference in effectiveness on purchase intention (Caputo et al., 2018). A third study compared the effects of providing GHG emissions information and water usage information in text form, finding that purchase intention was more affected by a water usage label (Grebitus et al., 2013). A fourth study examined the effects of a “pesticide-free” claim in text form compared to an organic claim in text form, finding that both had equal effects (Seo et al., 2019). Finally, a fifth study assessed combined organic (logo form) and water efficient claims (text form) compared with water efficient (in text form) only, and found the combined label was more effective (Wuepper et al., 2019).

Across these studies, evidence suggests that pesticide-free, organic, water use, and carbon footprint ecolabels are equally effective at changing consumer behavior. Compared with the ASC ecolabel, organic claims were more effective at changing behavior. Providing information about the GHG emissions of a product, regardless of the level of GHG emissions presented, was effective at changing behavior compared with control. The Rainforest Alliance Certified ecolabel was more effective compared to the carbon footprint ecolabel. Finally, making an ecolabel more accessible by providing a “total score” and color-coding increased its effectiveness.

### Differences by Demographic Groups: Gender, Age, and SES

Twenty studies assessed the impact of different socio-demographic characteristics on the effectiveness of ecolabels, with inconsistent findings.

Fifteen studies (10 hypothetical) examined the interaction between ecolabel effectiveness and participant gender. In ten studies (four real, six hypothetical), females were found to be influenced more positively than males by ecolabels (Aerni et al., 2011; Aguilar et al., 2010; Ankamah-Yeboah et al., 2018; Aoki et al., 2017; Blend & Van Ravenswaay, 1999; Campbell-Arvai et al., 2014; Cholette et al., 2013; Durham et al., 2012; Grunert et al., 2014; Wessells et al., 1999). In another four studies, of which one was conducted in a real setting, there was no observed interaction by gender (Brunner et al., 2018; Carlsson et al., 2010; Gumirakiza et al., 2017; Harwood & Drake, 2018). Finally, in one study (hypothetical), men were found to be more positively influenced by ecolabels (Shuai et al., 2014).

Nine studies (six hypothetical) showed mixed results regarding the impact of age. Four studies (one in real and three in hypothetical settings) reported that older consumers were more positively influenced by ecolabels (Ankamah-Yeboah et al., 2018; Aoki et al., 2017; Cholette et al., 2013; Grunert et al., 2014). Another four (again, one in real and three in hypothetical settings) found the reverse (Blend & Van Ravenswaay, 1999; Brunner et al., 2018; Durham et al., 2012; Shuai et al., 2014) and one study (real setting) did not show differences by age group (Aerni et al., 2011).

Twelve studies (10 hypothetical) examined differences in effectiveness of ecolabels based on participant income. Seven studies (two real, five hypothetical) showed ecolabels had positive effects of greater magnitude among participants with higher incomes (Aoki & Akai, 2013; Aoki et al., 2017; Cholette et al., 2013; Durham et al., 2012; Gumirakiza et al., 2017; Harwood & Drake, 2018; Shuai et al., 2014), two studies (hypothetical) showed stronger effects among participants with lower incomes (Ankamah-Yeboah et al., 2018; Delmas & Lessem, 2017), and three studies (hypothetical) showed no effect of income (Panzone et al., 2011; Rokka & Uusitalo, 2008; Wessells et al., 1999).

Nine studies (all hypothetical) examined the effects of ecolabels in relation to education level. Four studies showed that consumers with higher education were more likely to choose ecolabeled products (Blend & Van Ravenswaay, 1999; Durham et al., 2012; Harwood & Drake, 2018; Shuai et al., 2014), one study found that higher education decreased the likelihood of selecting an ecolabeled product (Delmas & Lessem, 2017), and four studies found no difference due to education level (Aoki et al., 2017; Panzone et al., 2011; Rokka & Uusitalo, 2008; Wessells et al., 1999).

## Discussion

### Summary of Main Findings

Sixty out of 76 interventions that tested the use of a variety of ecolabels reported a positive effect on the selection, purchase or consumption of more environmentally sustainable food and drink products. There was no clear indication that a particular label format (logo-only, text-only, or both) was more effective than another. While the majority of the included studies were conducted in hypothetical environments, there was clear evidence in favor of the intervention in studies conducted in both environments. Most studies analyzed the effect at a population-level. In sub-group analyses, there was modest evidence that ecolabels may be more effective among women and those of higher income or education, but the effects of age were mixed.

### Strengths and Limitations

This is the first systematic review and synthesis of evidence in this area. The strengths of this review include a robust search strategy based on a pre-registered protocol, employing gold-standard Cochrane methods (McKenzie & Brennan, 2019), and drawing on an established taxonomy to classify intervention components (information vs. claims) (Rayner et al., 2013). Our certainty in the evidence is limited by methodological issues in the primary studies, but reassuringly there was no evidence that studies at higher risk of bias were more likely to find positive outcomes. An important limitation is that 41 of 56 studies used hypothetical experimental designs and did not evaluate actual behavior in real-life environments. The majority were DCEs and focused on selection or purchase outcomes. Only one paper evaluated consumption outcomes (one actual and one hypothetical) and had equivocal outcomes. However, given selection and purchase are natural precursors to consumption, the evidence is likely to be relevant to consumption behavior. Unlike a real-world setting where ecolabel effectiveness is dependent on whether customers pay attention to the label, studies involving DCE designs force participant exposure to the label. It is therefore important to proceed with caution when drawing conclusions regarding the effectiveness of these labels in a real-world setting.

We did not include studies from grey literature or studies published in languages other than English, so there is a possibility that some relevant ecolabeling literature was not captured here. We cannot rule out publication bias which may be a particular issue in this area where studies are less likely to be pre-registered than in clinical research. In addition, the heterogeneity of study designs and outcomes precluded meta-analysis as well as estimates of specific effect sizes. Further, the studies included in this review provided scant information on moderators. Additionally, many of the tested labels in this review were not corroborated using a known certification scheme, so we are unable to tell if the effects from these labels are due to greenwashing. Many of the studies were at high risk of bias, often because of the possibility that participants could predict the aim of the experiments. Others did not include random sequence generation or allocation sequence concealment. While DCE studies allow for the order of presentation of ecolabels to be randomized across participants, future study designs could strengthen their methodology by adopting a randomized controlled trial approach and testing interventions in real-world environments.

### Implications of this Research

While this review was not embedded in a theoretical framework, the findings could be easily incorporated into a well-established method for characterizing behavior change interventions, the COM-B model. This framework proposes three essential conditions to enable behavior change: capability, opportunity, and motivation (Michie et al., 2014). Following this framework, providing environmental sustainability information (ecolabels) on products may motivate sustainable food selection, purchase or consumption. Psychological capability could be enhanced by ecolabels through educating shoppers on the environmental impacts of their food purchases. The opportunity to make swaps for more environmentally-friendly products would be increased if more products included environmental impact information at point-of-choice. Further, motivation to change shopping behavior could be increased by providing shoppers with nudges in-stores as well as through educational campaigns on the value of making these changes on the environment.

Indeed, ecolabels can provide consumers with information about the environmental credentials of their diet to facilitate informed choices, but there is no consistent ecolabel format and a paucity of evidence on which label may be most effective. There is tentative evidence of greater effectiveness if the ecolabel is backed by a certification scheme, implying that consumer trust in the credibility and validity of the label is important. These findings are consistent with findings regarding nutrition labeling, where evidence also suggests an effect but is mostly derived from hypothetical studies (Crockett et al., 2018). Most of the studies included in this review were designed to isolate the effect of ecolabeling. In practice, many other factors may influence the likelihood of selecting a product with an ecolabel, such as product price, product type, and awareness of the label itself (Littlewood et al., 2016). A review of grocery store interventions found that price had a significant effect on purchases (Hartmann-Boyce et al., 2018) and products with higher environmental standards are often offered at a price premium (Roheim et al., 2011). A companion review examines individuals’ willingness to pay for ecolabeled food products (Prospero ID: 42018094330). In a recent systematic review of 30 studies, consumers reported higher preference for ecolabel and social responsibility labels compared to nutrition labels (Tobi et al., 2019). This review concluded that a combination of environmental and social responsibility labels might be effective at increasing stated preference for products. It did not investigate effects on selection, purchase or consumption.

The present review is concerned with the effects of ecolabels on behavior and not whether the various labels are accurate representations. For example, sustainability is a core component of consumers’ perception of organic claims and is therefore relevant for this review, however, there is debate around whether organic farming methods are more sustainable than conventional methods (Leifeld, 2012; Tricase et al., 2018). Similarly, food and drink products that display GHG emissions labels may or may not be sustainably produced if other environmental indicators (e.g., land use, water use) for the product were calculated. However, all of these labels assessed here make implicit or explicit claims related to sustainability.

### Future Directions of this Research

Evidence from this review suggests that ecolabeling could be used to improve the likelihood of consumers selecting, purchasing and consuming more environmentally sustainable products. Defining what credentials a product should have to be awarded an ecolabel requires further research but will be important to ensure the credibility of such labels among the public. Future research needs to investigate the most effective type of label in changing consumer behavior and needs to assess whether the impact varies based on sociodemographic factors.

Crucially, more high-quality research is needed in real-world settings to enable more robust conclusions about the likely impact of ecolabels if adopted as a policy action. This includes the potential for unintended consequences, such as the effect of ecolabels on the purchasing of products that may have negative impacts on human health. The potential for a combined system of ecolabeling with nutrition labeling, or the use of ecolabels only on products meeting certain nutritional criteria, could be explored.

## Conclusion

This review provides preliminary evidence that ecolabels can promote the selection, purchase and consumption of more sustainable food and drinks. More high quality research is needed on the effectiveness of different ecolabel attributes and their effects in real world settings.

## Supplemental Material

sj-pdf-1-eab-10.1177_0013916521995473 – Supplemental material for The Effects of Environmental Sustainability Labels on Selection, Purchase, and Consumption of Food and Drink Products: A Systematic Review Click here for additional data file. Supplemental material, sj-pdf-1-eab-10.1177_0013916521995473 for The Effects of Environmental Sustainability Labels on Selection, Purchase, and Consumption of Food and Drink Products: A Systematic Review by Christina Potter, Anastasios Bastounis, Jamie Hartmann-Boyce, Cristina Stewart, Kerstin Frie, Kate Tudor, Filippo Bianchi, Emma Cartwright, Brian Cook, Mike Rayner and Susan A. Jebb in Environment and Behavior
