# Supplementary material for: The Effects of Environmental Sustainability Labels on Selection, Purchase, and Consumption of Food and Drink Products: A Systematic Review
Source: Environ Behav. 2021 Feb 20;53(8):891–925. doi: 10.1177/0013916521995473 (PMC8384304; doi:10.1177/0013916521995473)

Online Appendix

Supplemental Table 1. MEDLINE search strategy

# ▲	Searches
1	(ecolabel$ or eco-label$).ti,ab.
2	((environment$ or ecolog$ or eco or sustain$ or green$) adj5 label$).ti,ab.
3	((carbon footprint or recycl$ or organic$) adj5 label$).ti,ab.
4	1 or 2 or 3
5	Food Preferences/
6	Eating attitudes/
7	Feeding Behavior/
8	exp Eating/
9	Diets/
10	choice behavior/
11	(intak$ or consume or consumes or consumption or consumed or eat$ or diet$).ti,ab.
12	(food adj5 (preference$ or habit$ or behavio?r$ or choice$ or decision$ or decid$ or inclin$ or lik$ or choos$ or select$ or pick$)).ab,ti.
13	((drink? or beverage?) adj5 (preference$ or habit$ or behavio?r$ or choice$ or decision$ or decid$ or inclin$ or lik$ or choos$ or select$ or pick$)).ab,ti.
14	(purchas$ or buy$ or sale$ or vend$ or sell$).ab,ti.
15	Consumer behavior/
16	((willing$ or motivat$ or happy) adj5 (pay$ or spend$)).ti,ab.
17	5 or 6 or 7 or 8 or 9 or 10 or 11 or 12 or 13 or 14 or 15 or 16
18	4 and 17

Supplemental Figure 1. PRISMA flow diagram of study inclusion

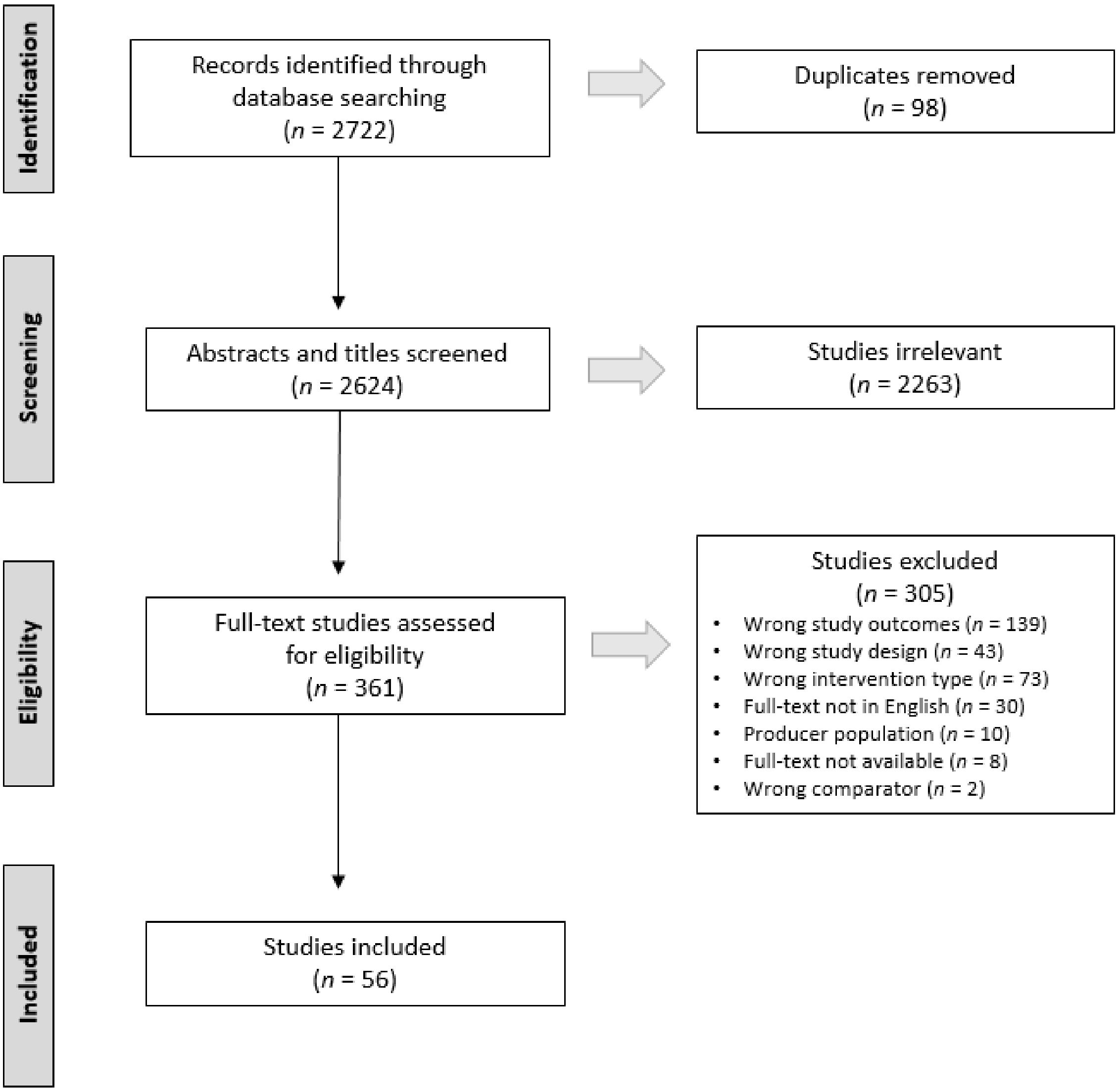

Supplement: sj-pdf-1-eab-10.1177_0013916521995473 – Supplemental material for The Effects of Environmental Sustainability Labels on Selection, Purchase, and Consumption of Food and Drink Products: A Systematic Review [file sj-pdf-1-eab-10.1177_0013916521995473.pdf]
